# Comparison of the subjective satisfaction of the donor site morbidity: Free radial forearm flap versus anterolateral thigh flap for reconstruction in tongue cancer patients

**DOI:** 10.4317/medoral.22679

**Published:** 2019-03

**Authors:** Li Meng, Jun Shen, Hao Liu, Jian-Cheng Zhang, Xin Peng, Chi Mao, Zhi-Gang Cai, Lei Zheng, Xiao-Feng Shan, Ying-Bin Yan

**Affiliations:** 1 BDS. Department of Oral and Maxillofacial Surgery, Tianjin Stomatological Hospital, 75 Dagu Road, Heping District, Tianjin 300041, PR China; 2 PhD. Department of Oral and Maxillofacial Surgery, Tianjin Stomatological Hospital, 75 Dagu Road, Heping District, Tianjin 300041, PR China; 3 MD. Department of Oral and Maxillofacial Surgery, Tianjin Stomatological Hospital, 75 Dagu Road, Heping District, Tianjin 300041, PR China; 4 MD. Department of Oral and Maxillofacial Surgery, Peking University School and Hospital of Stomatology, 22 Zhongguancun Nandajie, Haidian district, Beijing 100081, PR China; 5 BDS, MDS, PhD. Department of Oral and Maxillofacial Surgery, Tianjin Stomatological Hospital, 75 Dagu Road, Heping District, Tianjin 300041, PR China

## Abstract

**Background:**

The purpose of the study was to compare the differences of the subjective satisfaction of the donor site morbidity between the free radial forearm flap (FRFF) and anterolateral thigh flap (ALTF) for tongue reconstruction.

**Material and Methods:**

One hundred and nineteen patients underwent FRFF or ALTF reconstruction were retrospectively evaluated by a standardized self-established donor site morbidity questionnaire which included 5 domains, sensibility, movement disabilities, cosmetics, social activities and general impacts on the quality of life.

**Results:**

The Cronbach’s coefficient alpha of the questionnaire was 0.707. The exploratory factor analysis revealed that the 5 items of the questionnaire might load onto two distinct subscales. Patients with ALTF had higher scores in the sensibility, cosmetics and the composite score (*P*< 0.05). No significant differences were found in the movement disabilities, social activities and general impacts on the quality of life between the two groups (*P* > 0.05).

**Conclusions:**

ALTF has the advantage of better results of donor site morbidity, such as sensibility and cosmetics, over FRFF.

** Key words:**Quality of life, tongue cancer, free radial forearm flap, anterolateral thigh flap, donor site morbidity.

## Introduction

Free radial forearm flap (FRFF) and anterolateral thigh flap (ALTF) have established themselves as the most popular choices for reconstruction of tongue and other intra-oral defects. However which flap is better remains controversial. The most advantage of ALTF over FRFF may be at the donor site. With the ALTF, the defect at the donor site is almost always closed primarily, however harvesting FRFF leaves a large defect requiring skin grafting.

Some studies had revealed that patients reconstructed with the ALTF had lower complications, better functional results and aesthetic outcomes at donor site than those with FRFF by objective evaluation (1-6). However, few studies had reported on the patients’ subjective views of the donor site satisfaction (7,8). The inconsistency between the subjective and objective testing had also been shown in several studies on the donor site morbidity of FRFF (9-11).

 Despite the importance of the previous studies on the subjective evaluation of the donor site morbidity (7,8), lack of a standardized questionnaire will impede the comparison between different studies. Therefore, the aim of this retrospective study was to compare the differences of the donor site morbidity, between the FRFF and ALTF for tongue reconstruction, by a standardized self-established scale.

## Material and Methods

-Patients

Two tertiary stomatological hospitals, Tianjin Stomatological Hospital and Peking University School and Hospital of Stomatology, were included. From January 2011 to January 2016, a total of 250 consecutive patients underwent FRFF or ALTF reconstruction after ablative surgery for untreated, primary tongue squamous cell carcinoma (ICD-10, C02 and C01.9) at the studied hospitals. Patients who received surgery due to a recurrent disease, had been irradiated before surgery, or had inadequate medical records were excluded. Patients who were with malignant tumor in other body regions or with flap loss were also excluded.

From October 2016 to December 2017, the patients were asked to answer a donor site morbidity questionnaire by the same trained doctor through a telephone interview. The doctor completed the questionnaire by directly reading the questions to the patients and recording their verbal responses. The local ethics committees approved the retrospective study. Of the 250 patients, 76 (30.4%) died because of cancer or other diseases, 42 (16.8%) lost to contact, 8 (3.2%) refused to answer the questionnaires, 5 (2%) were excluded because of recurrence after reconstruction surgery, and 119 (47.6%) completed the questionnaires.

Sociodemographic data (gender, age, education level), comorbidity (cardiovascular disease, diabetes mellitus, pulmonary problems, or other system disorders), and clinical data (tumor stage, extent of tongue resection, the flap size, the modes of tongue reconstruction, and follow-up period) were retrospectively gathered from the medical records. The extent of tongue resection were divided into two categories: hemiglossectomy (half the tongue), and subtotal glossectomy (more than half but less than three quarters of the tongue). The modes of tongue reconstruction were divided into FRFF group and ALTF group.

-The donor site morbidity questionnaire

The donor site morbidity questionnaire was a self-established scale which comprised 5 items: 4 are disease-specific (sensibility, movement disabilities, cosmetics, and social activities) and 1 is a general question ( Table 1). Each of the questions has 5 response options: “not at all”, “a little bit”, “somewhat”, “quite a bit”, and “very much”. The domains are scored on a scale ranging from 0 (“very much”) to 100 (“not at all”) using a 5-point Likert type scale. A composite score from 0 to 100 was obtained by averaging the scores of the domains. The higher scores indicate better quality of life.

**Table 1:**
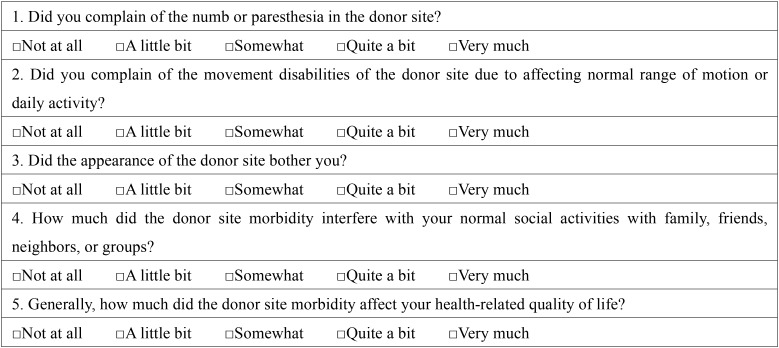
The donor site morbidity questionnaire: this questionnaire asks patients about their quality of life over the past 7 days.

-Statistical methods

Internal consistency reliability was measured by Cronbach’s coefficient alpha. If an item fails to correlate well with the other items, the Cronbach’s alpha increases in its absence. The construct validity was evaluated by exploratory factor analysis, grouping individual questions with strong correlation into discrete cluster or constructs. A conventional varimax method of rotation was used.

For descriptive purposes, mean and standard deviation (SD), and median and range were used. The independent t tests or Chi-square tests were used to explore any difference in patient’s characteristics between the FRFF group and ALTF group. The Mann-Whitney U-test was used to compare differences of the donor site morbidity between the FRFF group and ALTF group.

SPSS 17.0 software package (SPSS Inc., Chicago, IL) for Windows was used for the statistical analysis. The level of statistical significance was taken as *P* < .05.

## Results

-Patients characteristics

Patients’ characteristics were shown in  Table 2. The mean follow-up time was 37.1 months (range 23-76). Of these, 75 (63.1%) were male and 44 (36.9%) were women; overall mean age at the time of operation was 53.0 years (range 25-78).

**Table 2:**
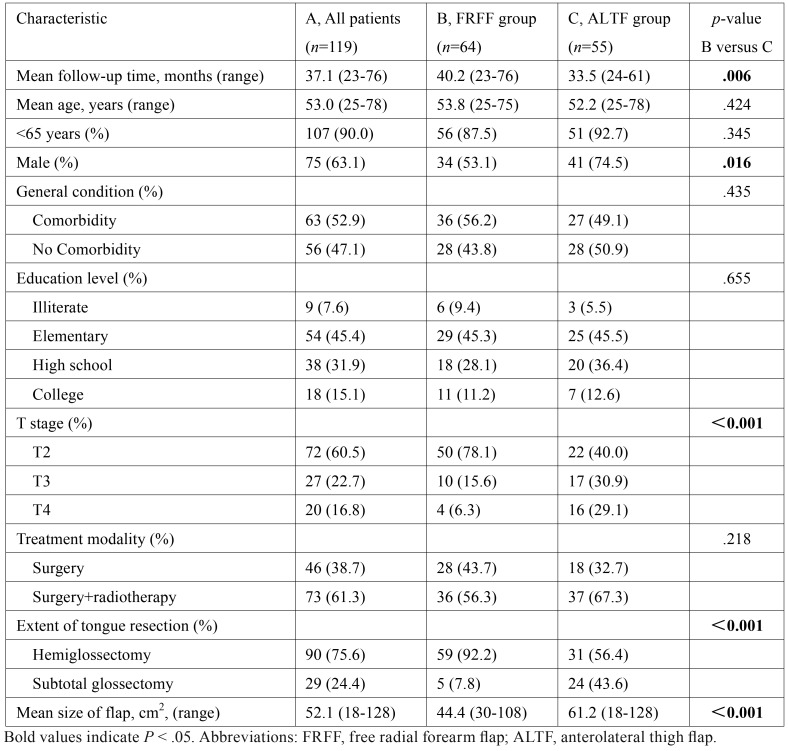
Descriptive and bivariate analyses of tongue cancer patients who underwent FRFF or ALTF reconstruction.

Of the 119 responders, 90 (75.6%) underwent hemiglossectomy and 29 (24.4%) subtotal glossectomy. FRFF was performed in 64 (53.8%) and ALTF in 55 (46.2%) patients. All patients reconstructed with the FRFF had split-thickness skin graft, while the donor defects of the ALTF were closed directly. There were no significant differences in age, comorbidity, education level and postoperative radiotherapy between the FRFF group and ALTF group (see  Table 2, B versus C).

The mean follow-up time in FRFF group (40.2 months) was longer than that in ALTF group (33.5 months) (*P* = .006). The difference in the proportions of man between the two groups was statistically significant (*P* = .016). Compared to ALTF group, patients in FRFF group had a significantly lower disease stage (*P* < .001), received a significantly smaller extent of glossectomy (*P* < .001) and a significantly smaller flap size (*P* < .001) (see  Table 2, B versus C).

-The reliability and construct validity of the donor site morbidity questionnaire

**Figure 1:**
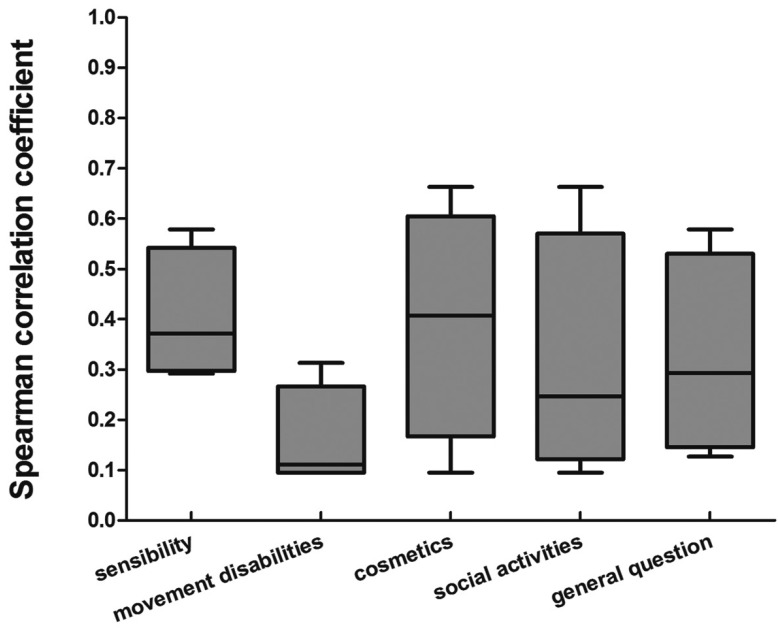
Inter-item correlations for the donor site morbidity questionnaire. Each box and whisker is a summary of 4 correlations of the item with the other 4 items.

The Cronbach’s coefficient alpha of the questionnaire was 0.707. The loss of any single item did not change the alpha coefficient to any great extent (range, 0.578-0.741). The Cronbach’s alpha decreased if an item was deleted, except for the item of movement disabilities. All the inter-item correlations were positive and were shown for each item in figure 1. The movement disabilities correlated less with other items.

The principal components factor analysis extracted two principal components (the initial eigenvalues > 1) from the 5 items (see  Table 3). The analysis explained 68.9% of the total variation, with the first factor accounting for 36.4% and the second factor 32.5%. The items that loaded more strongly (loadings ≥ 0.40) onto the first factor were cosmetics (0.879) and social activities (0.878). The second factor was made up of sensibility (0.782), movement disabilities (0.721), and generel question (0.667).

**Table 3:**
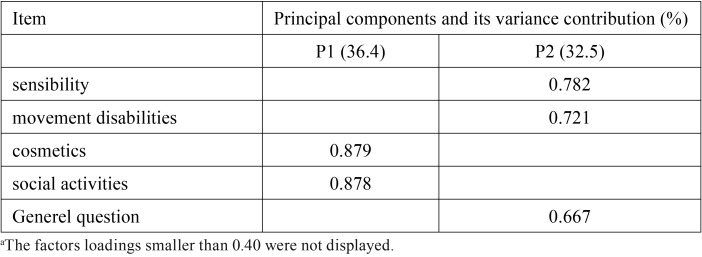
The two principal components and factor loadings of their related items (n = 119)a.

-The differences of subjective satisfaction of the donor site morbidity

The scores for the five domains and composite score were demonstrated in  Table 4. The differences between groups were statistically significant for sensibility (89.4 ± 23.6 vs. 97.7 ± 8.8, *P* = .025), cosmetics (74.1 ± 31.8 vs. 85.2 ± 25.5, *P* = .048), and composite score (86.9 ± 17.1 vs. 93.3 ± 9.3, *P* = .018), while patients in ALTF group had more favorable scores. No significant difference was found in the movement disabilities, social activities and general question domains between the two groups, although the ALTF group was inclined to obtain higher scores for social activities (87.5 ± 25.6 vs. 93.5 ± 15.5, *P* = .444) and general question (84.3 ± 24.4 vs. 91.2 ± 18.3, *P* = .141).

**Table 4:**
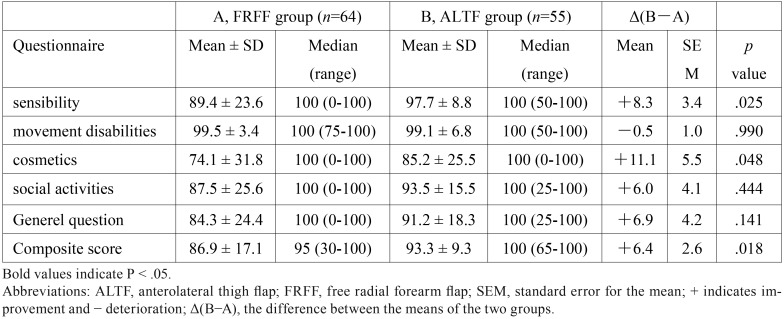
The scores of the donor site morbidity questionnaire of tongue cancer patients underwent different modes of reconstruction.

## Discussion

The important goal of reconstructive surgery in tongue cancer patients is to maximally recover the oral function. In addition, the donor site morbidity after the flap harvest is another important issue when tailoring a suitable flap. Although the use of objective measures of the donor site morbidity provided important information, the use of a questionnaire to evaluate the subjective complaints from a patient perspective could not be neglected, especially when the inconsistency between the subjective and objective testing existed.

In the present study, we designed a standardized questionnaire to measure the subjective satisfaction of the donor site morbidity after harvesting ALTF or FRFF. The psychometric properties, such as reliability and construct validity, were evaluated. Internal consistency reliability was one of most frequently used methods for the evaluation of the instrument reliability. It was recognized that the Cronbach’s coefficient alpha should be at least 0.70 (12). Our result showed that the Cronbach’s coefficient alpha was a little greater than 0.7, indicating reasonable reliability. Factor analysis was performed to assess the construct validity of the instrument. The results of the exploratory factor analysis suggested a couple of things: first that the 5 items of the questionnaire might load onto two distinct subscales (factors) and second, the cosmetics had an impact on the social activities of the patients, while the sensibility and movement disabilities affected the overall health-related quality of life.

Data obtained in the present study demonstrated that donor site sensibility, cosmetics, and the composite score of the questionnaire were better when ALTF was used. Harvest of FRFF may lead to diminished sensation supplied by antebranchial cutaneous nerve, with the rate of abnormal sensitivity ranging from 26% to 80% (2,7,9,13). As a comparison, the incidence of lateral thigh paresthesia for ALTF was only 24% due to the injured or sacrificed lateral cutaneous nerve of the thigh (14). Therefore, the incidence of sensory abnormalities in the donor site of ALTF was lower than that in FRFF. Accordingly, the different subjective feelings between the two groups were also measured by the questionnaire.

For the forearm donor site, a split- or full-thickness skin graft is often necessary, although direct closure can be achieved by an ulnar fasciocutaneous V-Y transposition flap for defects smaller than 6×4 cm (1). However, the two methods result in large donor scar which may disturb the patients. On the contrary, the donor site of ALTF can be closed primarily in most patients unless the width of defect is > 8 cm. The scar left on the thigh is often obvious but relatively hidden, allowing patients to easily accept it. Although patients with FRFF could accept and well tolerated the appearance at the donor site (10), the superiority of ALTF was revealed in the present study which was in accordance with several previous reports (2,7,8,15).

Movement disability of forearm was another serious complication of FRFF. Although some studies described rare or no movement disabilities after harvest of FRFF (7,16), others reported a reduced wrist mobility, reduced strength, or hand dexterity by objective testing (2,8,9). In the present study, the scores of movement disabilities in the FRFF group were 99.5 ± 3.4, indicating no movement disabilities were complained by patients. We also found no significant difference in the movement disabilities at the donor site between the two groups. Similar results were reported by Huang *et al.* (7) who found no patient with FRFF or ALTF complained of the donor site affecting normal daily activity. In contrast, Cigna *et al.* (8) found that manual dexterity was slower on the operated donor site than on the non-operated side in the 33.3% of FRFF patients, while the function of the knee of the ALTF patients was the same in the donor site and non-donor. However, since the non-dominant arm was often selected as the donor arm, the difference of the manual dexterity between the operated donor site and the non-operated side may be the result of use of dominant hand (8). In addition, it should be noted that there was a difference between the objective and subjective evaluations of the movement disabilities (9,10), and the measurable quantitative changes in hand function may only result in limited or no changes in patient perception (16).

In the present study, we found the trend of better social activities in the ALTF group, but no significant difference. Better social function was revealed when the ALTF was used (8). Ten percent of FRFF patients considered the donor site as a significant negative social stigma affecting the quality of life, while none of ALTF patients thought so (7). Li *et al.* (15) found that patients that underwent FRFF reported lower scores of social function than those with ALTF by the Medical Outcomes Study-Short Form-36 (SF-36), indicating that the donor site scar might affect patients’ normal social activities. An important reason why our results were different with the abovementioned studies might lie in the differences between Chinese and Western cultures. In addition, the measure of social activities was different. We used the donor site specific scale, and only the impact of the donor site morbidity on the social activities was measured. However in the studies of Li *et al.* (15) and Huang *et al.* (7) both the recipient and donor site morbidity were involved.

Several factors required attention when interpreting the findings of the study. First, the cross-sectional design allowed for rapid evaluation of the differences of donor site morbidity, but the inaccuracy of clinical parameters was an inherent flaw. Second, due to the designed drawback, we did not perform a second assessment in a short time interval, so the test-retest reliability, another important reliability index, could not be calculated. Third, it was not a longitudinal study, and the responsiveness of the questionnaire over time could not be explored, although the present study revealed that the instrument could detect the differences of donor site morbidity between groups sensitively.

## Conclusions

Data obtained in the present study showed that ALTF has the advantage of better results of donor site morbidity, such as sensibility and cosmetics, over FRFF.
